# Significantly reduced radiation dose to operators during percutaneous vertebroplasty using a new cement delivery device

**DOI:** 10.1186/1471-2474-15-260

**Published:** 2014-08-01

**Authors:** Guang-Quan Zhang, Yan-Zheng Gao, Shu-Lian Chen, Shuai Ding, Kun Gao

**Affiliations:** 1 Orthopedic of henan provincial people’s hospital(Zhengzhou university people’s hospital), Weiwu road 7, Zhengzhou city, henan province 450003, China

**Keywords:** Percutaneous vertebroplasty, Cement delivery device, Radiation dose

## Abstract

**Background:**

Percutaneous vertebroplasy (PVP) might lead to significant radiation exposure to patients, operators, and operating room personnel. Therefore, radiaton exposure is a concern. The aim of this study was to present a remote control cement delivery device and study whether it can reduce dose exposue to operators.

**Methods:**

After meticulous preoperative preparation, a series of 40 osteoporosis patients were treated with unilateral approach PVP using the new cement delivery divice. We compared levels of fluoroscopic exposure to operator standing on different places during operation. group A: operator stood about 4 meters away from X-ray tube behind the lead sheet. group B: operator stood adjacent to patient as using conventional manual cement delivery device.

**Results:**

During whole operation process, radiation dose to the operator (group A) was 0.10 ± 0.03 (0.07-0.15) μSv, group B was 12.09 ± 4.67 (10–20) μSv. a difference that was found to be statistically significant (P < 0.001) between group A and group B.

**Conclusion:**

New cement delivery device plus meticulous preoperative preparation can significantly decrease radiation dose to operators.

## Background

Percutaneous vertebroplasy (PVP) is highly dependent upon intraoperative fluoroscopic visualization, which might lead to significant radiation exposure to patents, operators, and operating room personnel. therefore, radiaton exposure is a concern. While performing fluoroscopic procedures, the operator should continuously minimize radiation dosage by using all reasonable methods. This principle is referred to as “ALARA (As Low As Reasonably Achievable)”. Time, Distance, and Shielding are three major techniques employed to maintain ALARA dosages [1]. When the operator performs PVP with conventional manual cement delivery device, he has to stand beside the patient and can not be far away from the X ray tube. A new cement delivery device, remote control cement delivery device, designed by guanlong company of China can increase the distance from the x ray tube. The operator standing behind the lead sheet a few metres away from the patient can remote control cement injection.

The purpose of our study was to compare the radiation exposure doses to an operator performing PVP with conventional manual cement delivery device and the new cement delivery device and to assess whether the new delivery device can reduce radiation exposure.

## Methods

### Subjects

**Figure 1:**
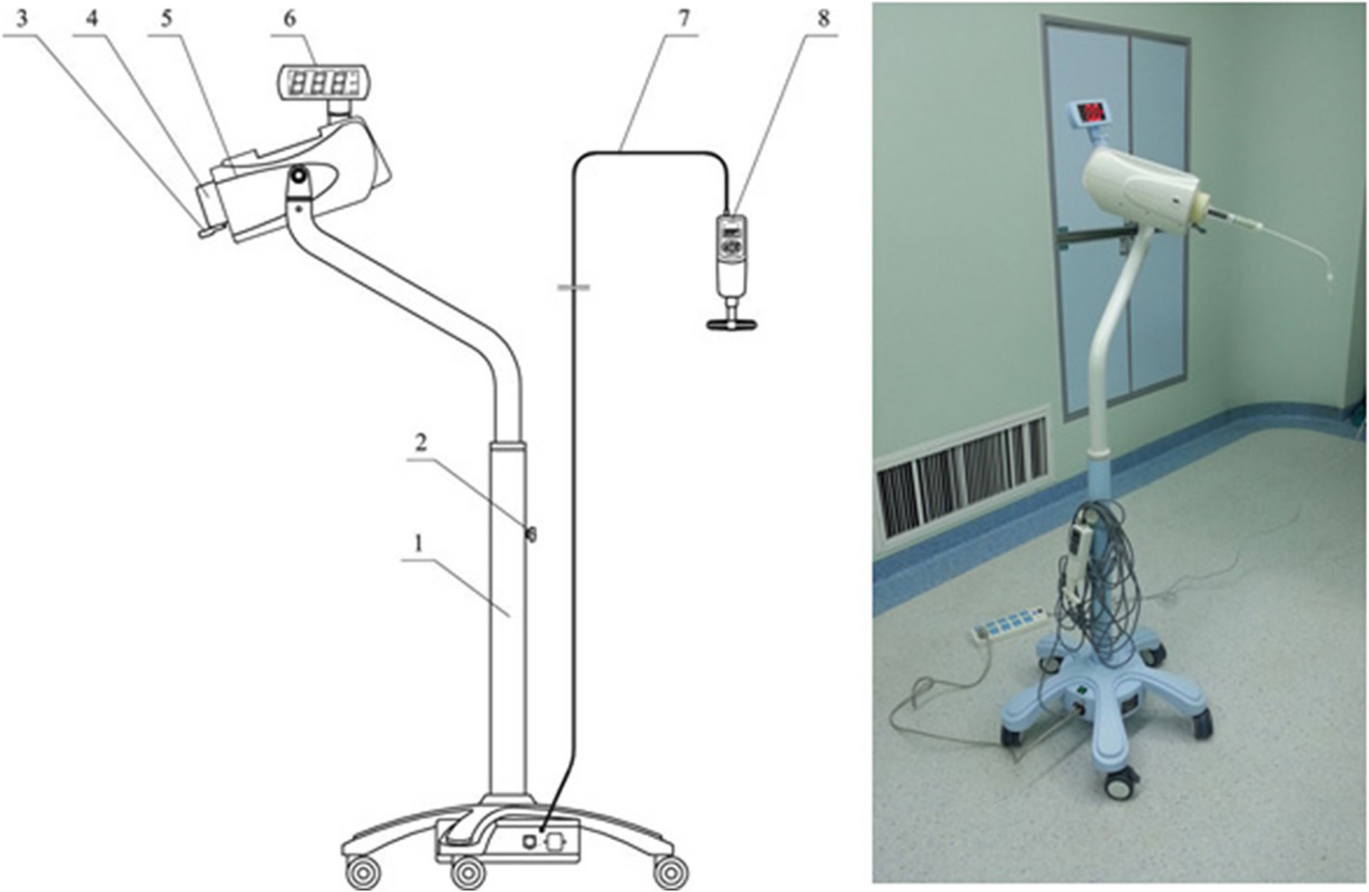
1 lifting bracket 2 lifting locking handwheel 3 locking handle4 clamping device5 control box 6 display 7 handheld controller cable (12 metres length) 8 handheld controller.

The cohort consisted of 40 patients with osteoporotic fracture, 8 males and 32 females, 65–86 years old (average, 76 years), admitted to Henan Province People’s Hospital between December 2012 and January 2014. The mean bone mineral density was −2.85 standard deviation (SD). The treated vertebrae were as follows: T4 (n = 1), T5 (n = 1), T7 (n = 2), T8 (n = 1), T9(n = 2),T10 (n = 5), T11 (n = 6), T12 (n = 6), L1 (n = 7), L2 (n = 5), L3 (n = 2), L4 (n = 1), L5 (n = 1). This study was approved by the Ethics Committee of the Henan Province People’s Hospital. Written informed consent was obtained from all participants. This manuscript has adhered to the STROBE guidelines (Additional file 1).Remote control cement delivery device was made in Guanlong company of China. The main structure is shown in Figure 1. When injection of cement, the pusing rod tail of propeller is inserted into the clamping device and stuck by the locking handle, the other end of propeller (the connecting pipe) is connected to the needle, the operator can stand behind the lead sheet as far as 12 meters away from X-ray tube and inject bone cement through the forward key on the handheld controller, but in our study, we stood behind the lead sheet 4 meters away.

### Dosimetry

**Figure 2:**
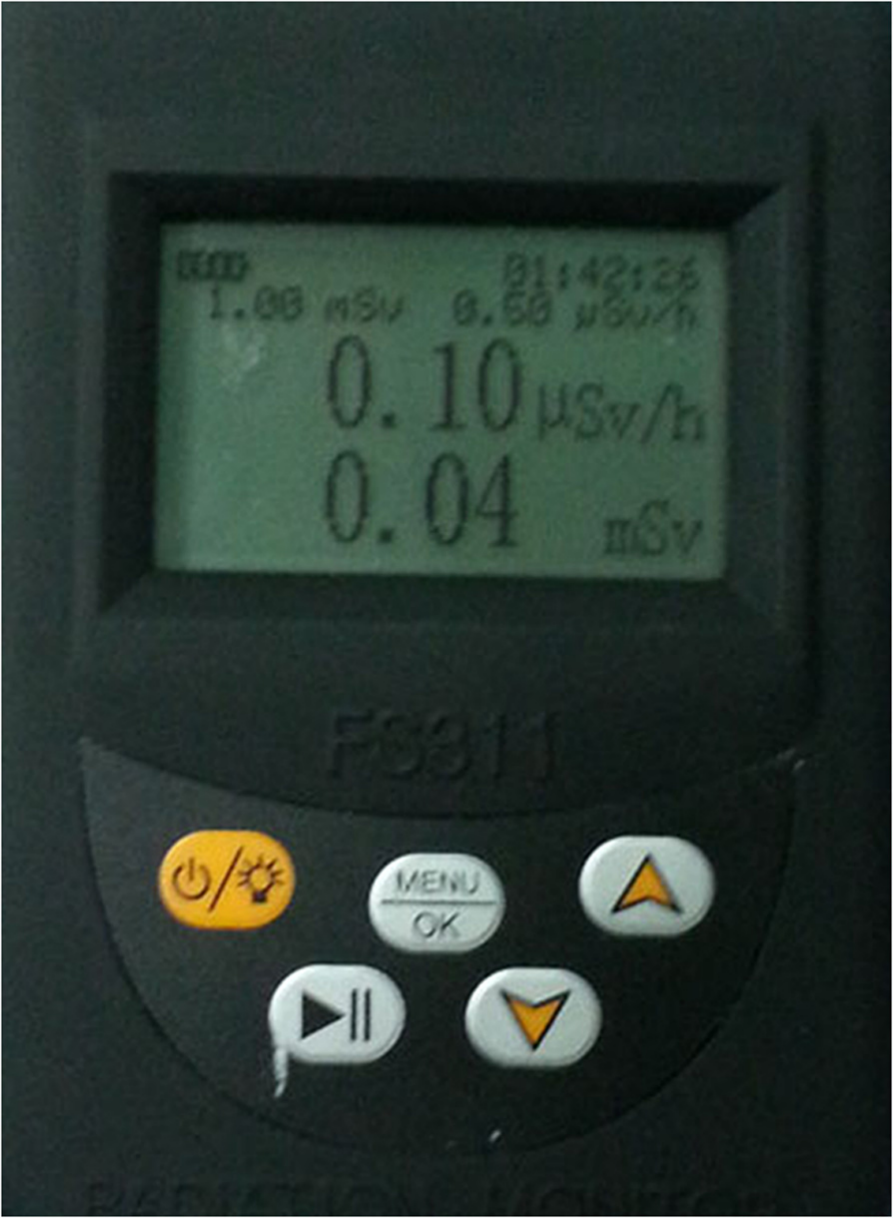
Thermoluminescent dosimeters(Beilite FS311, Wenzhou, China).

The position of the tube of the C-arm is below the operating bed when anterior posterior fluoroscopy, When lateral fluoroscopy, operator stand opposite to the tube. Thermoluminescent dosemeters (Beilite FS311, Wenzhou, China) (Figure 2) were placed on the right flank of each patient (adjacent to the affected vertebra) and the upper sternum (juxta-thyroid) of the operator (the group A) and the drip stand the same height as the operator’s upper sternum during operation. The drip stand is placed beside the patient as the place the operator standing when using conventional manual cement delivery divice (the group B, Simulation of the doctor standing beside the patient)opposite to the x-ray tube. Ideally, using two dosemeters in either plane, we can measure patient radiation exposure in both planes. However, it was impossible in the current study because the dosimeter is not sterile, which can not be placed in the surgical field. In general, lateral view should be taken more times than anteroposterior radiograph to check cement leakage into spinal canal [2]. Thus, in our study, we placed the dosimeter on the right flank of patient opposite to the x-ray tube.

### PVP technique

**Figure 3:**
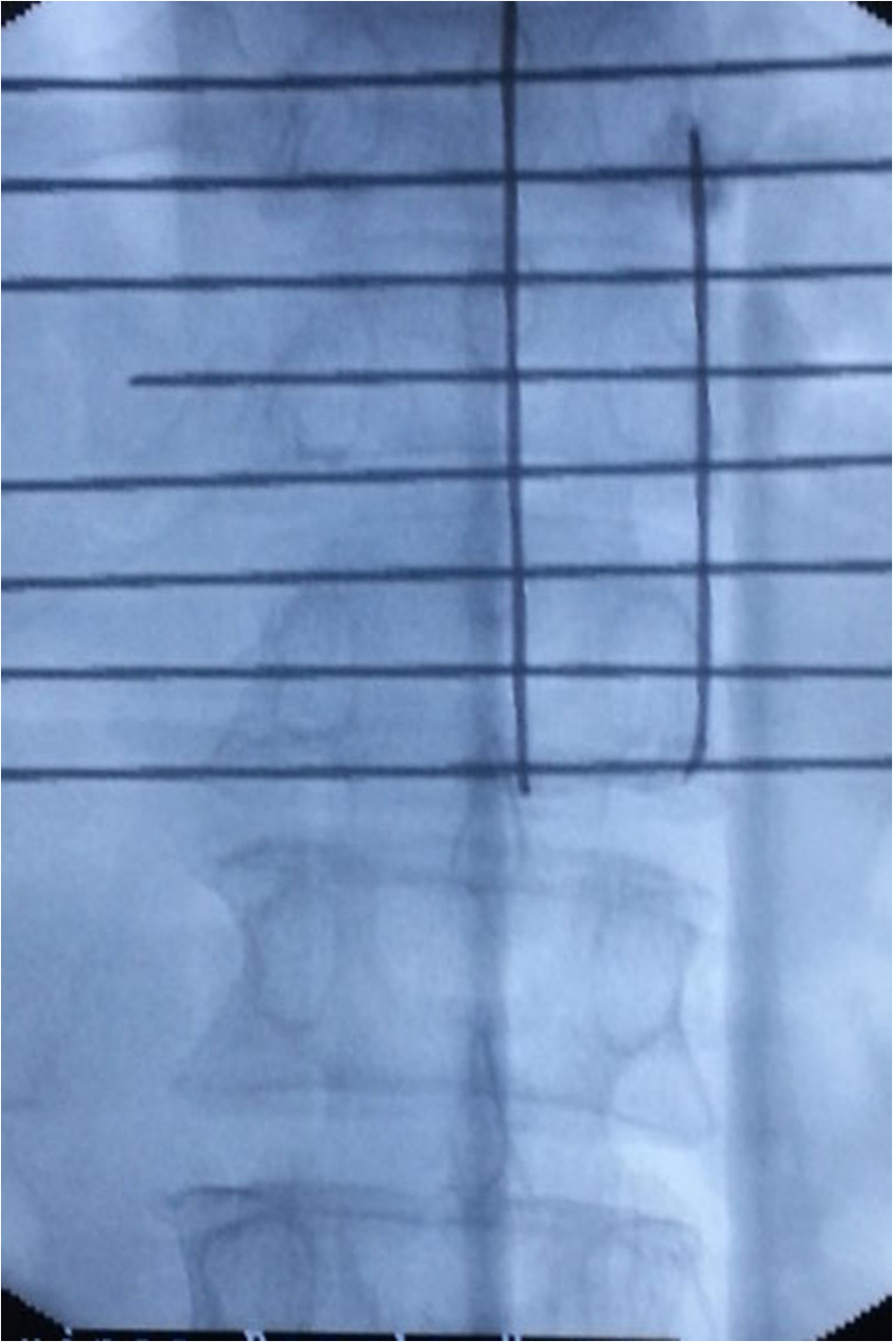
Using the self-made multiple grid device, we can position the affected vertebral body with lower radiation exposure.

**Figure 4:**
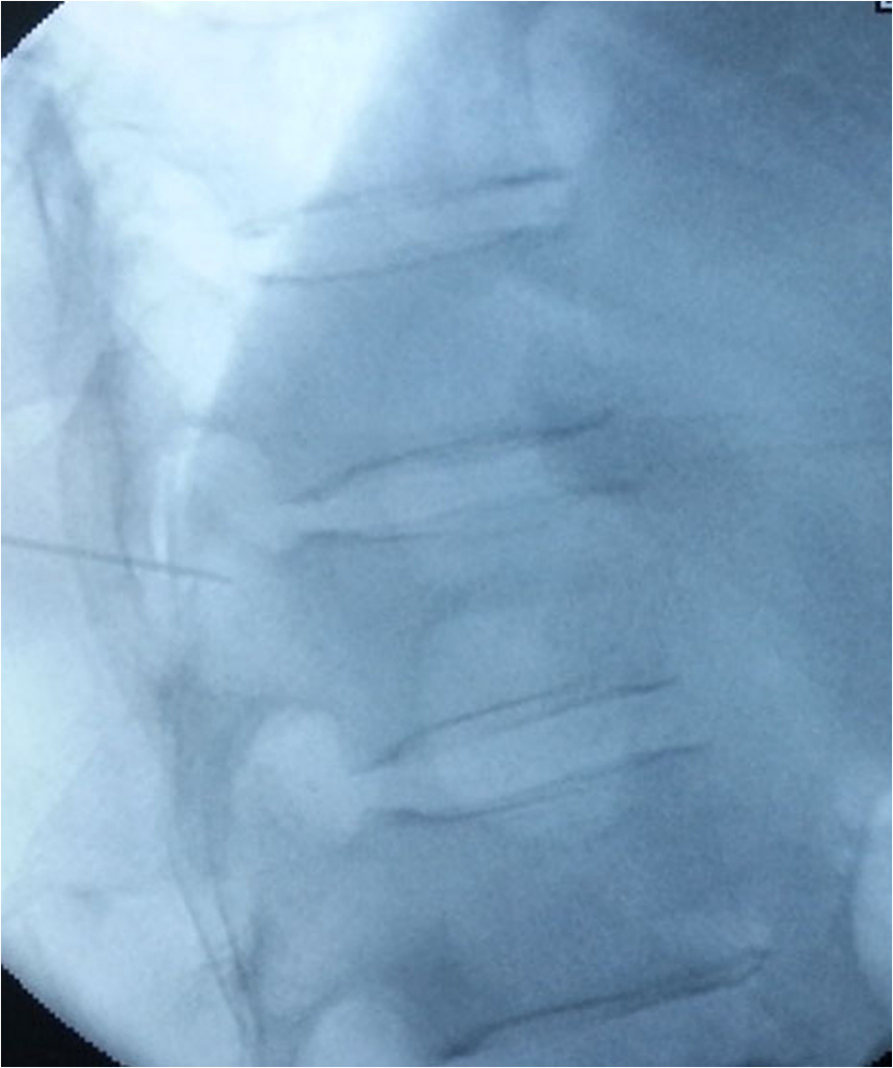
The 18G needle according to preoperative measurement of angle reached the parapedicle(at the same time, local anesthesia with 1% lidocaine is infiltrated into the skin and periosteum of the pedicle).

**Figure 5:**
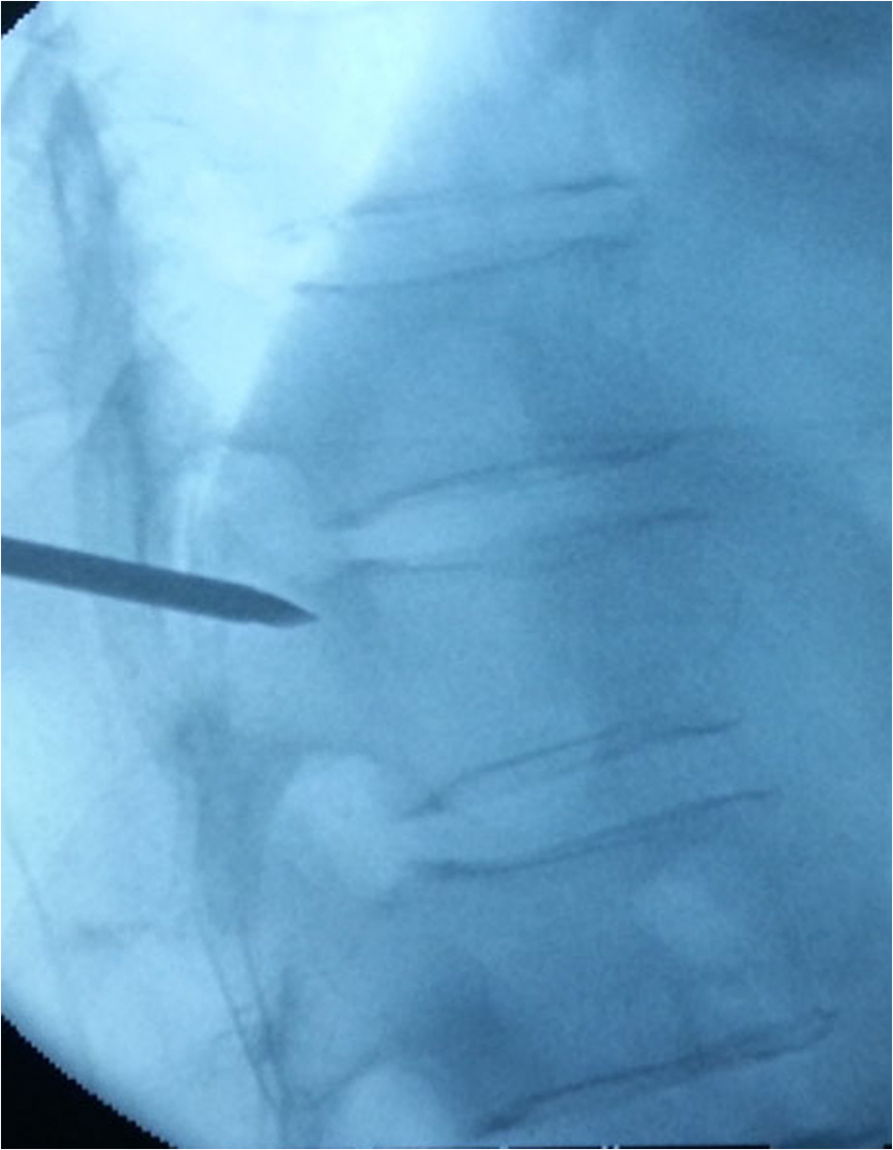
3.2 mm (130 mm length) diameter vertebral needle punctured by unilateral parapedicle approach along the direction of 18G needle.

**Figure 6:**
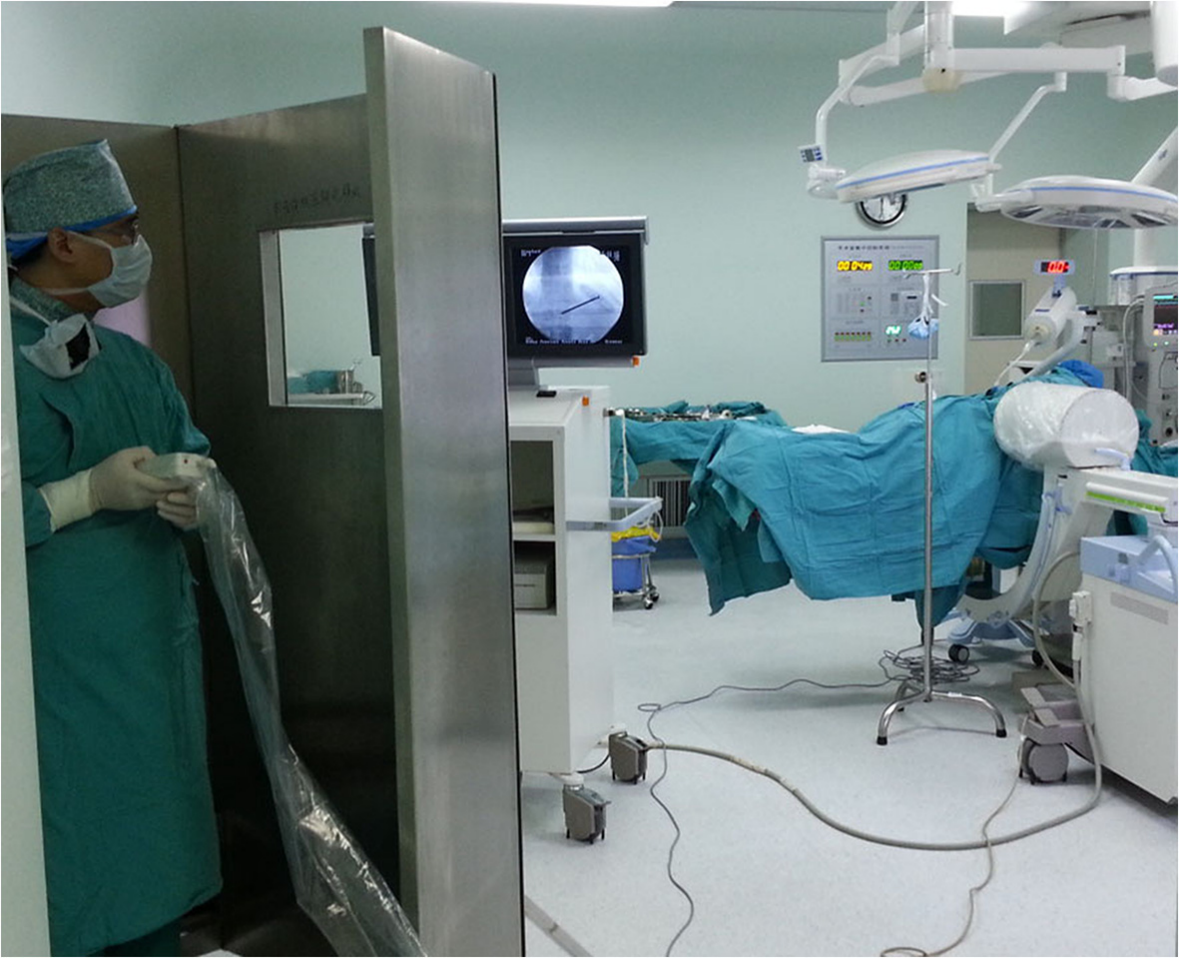
The operator stood behind the lead sheet about 4 meters away from X-ray tube and injected cement through the forward key on the handheld controller (it can be wraped by steriled sheath).

The patients were placed in the prone position and operated on local anesthesia. Pillows were used to support the upper chest and pelvis to enable maximum extension of the spinal column. This postural reduction generally restored some of the height of the fractured vertebrae. We used pedicle approach or parapedicle approach. According to the iliac crest, Twelfth rib et al. anatomic landmark, we initial positioned affected vertebral body, and then put the self-made multiple grid device on the above position, by fluoroscopy, we can accurate position the affected vertebral body (Figure 3). According to preoperative CT or MRI measurement, we got the distance between entry point and the midline. From the entry point, the 18G needle according to preoperative measurement of angle reached the pedicle or parapedicle(at the same time, local anesthesia with 1% lidocaine is infiltrated into the skin and periosteum of the pedicle). a skin incision about 0.5 cm was made, a 3.2 mm (130 mm length)diameter vertebral needle(guanlong, China) punctured by unilateral pedicle or parapedicle approach(Figures 4 and 5), Under guidance of fluoroscopic view, The vertebral needle reached the vertebral body between its anterior 1⁄4 and anterior 1⁄3, as seen on the lateral view, and to the midline of the vertebral body as seen on the AP view. the pusing rod tail of propeller is inserted into the clamping device and stuck by the locking handle, the other end of propeller is connected to the needle by the connecting pipe, the operator stood behind the lead sheet about 4 meters away from X-ray tube and injected cement through the forward key on the handheld controller (it can be wraped by steriled sheath) (Figure 6) (the operator Dr guangquan zhang has consent to publish the image).

The total operative time was recorded from the start of the local anesthesia to removal of the vertebral needle. The needle positioning time was from the start of anesthesia to finish positioning the spinal needle. The radiation dose (in μSv) and time (in min)to the operator and patient were measured during neele positioning and whole operation.

We compared the radiation dose to the operator during needle positioning and whole operation between group A and group B. For statistical evaluation the paired samples *T* test was performed using the SPSS 17.0 statistical software (SPSS, Inc., Chicago, IL, USA). P < 0.05 was considered to indicate a statistically significant difference.

## Results

The total operative time was 31.00 ± 7.90 (20–42) min, the needle positioning time was 16.30 ± 8.12 (5–28) min. the total fluoroscopy time was 0.78 ± 0.24 (0.5-1.1) min, the fluoroscopy time for needle positioning was 0.20 ± 0.12 (0.1-0.4) min.

During needle positioning, radiation dose to the patient was 6.65 ± 3.0 (2.74-10) μSv. during whole operative process, radiation dose to the patient was 25.5 ± 4.61 (20–30) μSv.

During needle positioning, radiation dose to the operator (group A) was 0.07 ± 0.03 (0.04-0.12) μSv, group B was 3.68 ± 2.87 (2.06-8.52) μSv. during whole operative process, radiation to the group A was 0.10 ± 0.03 (0.07-0.15) μSv, the group B was 12.09 ± 4.67 (10–20) μSv. a difference that was found to be statistically significant (P < 0.001) between group A and group B, not only in needle positioning but also in whole operation process.

## Discussion

Percutaneous vertebroplasty or kyphoplasty requires radiographic visualization in two planes (anteroposterior and lateral view) to identify the position of the spinal needle and real-time fluoroscopic monitoring is usually recommended during cement injection. a drawback of this technique is an important radiation exposure to the surgeon and patient. With an increasing number of vertebroplasty or kyphoplasty procedures being performed, the question about the importance of this radiation exposure for a surgeon and patient arose. Several studies have investigated the patient’s and the surgeon’s radiation exposure during vertebroplasty or kyphoplasy and found that the radiation-ralated risk may be considerable [2-4]. Adverse effects of ionizing radiation exposure to the human body are largely divided into two type. The early effects include acute radiation lethality, local tissue damage for skin or gonads, hematologic effects, and cytogenetic effects. The late effects include radiation-induced malignancy, such as leukemia and other forms of cancer, deleterious local issue effects, chromosomal toxicity, and/or cataractormation [3]. Theoretically the following technical aspects influence occupational radiation exposure of the surgeon: exposure time, distance, and shielding.

First, ideally, to reduce occupational radiation exposure, the total fluoroscopy time should be kept to a minimum. Boszczyk et al. [5] and Li et al. [2] reported the radiation exposure time of kyphoplasty using the two-fluoroscopic technique was shorter compared to other studies using the one fluoroscopic technique. Unfortunately, it would also make the surgeon’s work space narrow and increase the non-surgical phase of the procedure due to the initial time required to correctely position the two C-arms. Izadpanah K et al. [6] reported that navigation-guided kyphoplasty can reduce the radiaton exposure to operators. In the conventional kyphoplasty group, the average radiation times for thoracic spine (ts) and lumbar spine (ls) were 175 and 165 seconds. The average radiation time in the navigated group was reduced significantly in the navigated group (99 seconds ts and 74 seconds ls). However, several problems exists, such as the high cost of the equipment; as with other new intraoperative techniques, there is a relevant learning curve; an additional incision has to be performed, in order to attach the reference clamp. Ortiz et al. [7] performed 189 consecutive vertebral augmentation procedures in 135 patients with osteoporotic compression fractures by using a bilateral approach. A total of 87 kyphoplasty procedures, 82 vertebroplasty procedures with a cement delivery system CDS (VP-CDS), and 20 vertebroplasty procedures with syringes (VP-S) were safely performed. Mean fluoroscopy time for device positioning was 4.3 minutes for each procedure type. Mean fluoroscopy time (minutes) for cement delivery was significantly different for the 3 procedure types; 2.1 for kyphoplasty, 3.7 for VP-CDS, and 1.5 for VP-S. In our study, we used one-fluoroscopic technique, the total fluoroscopy time was only 0.78 min, fluoroscopic time for device positioning was only 0.20 min, we use methods as follows, First, to achieve the purpose of unilateral puncture operation, this can reduce the exposure time. We should have meticulous preoperative preparation. According to preoperative CT or MRI, we got the distance from entry point to the midline and puncturing angle. Secondly, According to the iliac crest, Twelfth rib et al. anatomic landmark, we initial positioned affected vertebral body, and then put the self-made multiple grid device on the above position, by fluoroscopy, we can accurate position the affected vertebral body. We can draw the midline, pedicle position and entry point. the 18G needle according to preoperative measurement of angle reached the pedicle or parapedicle (at the same time, local anesthesia with 1% lidocaine is infiltrated into the skin and periosteum of the pedicle). a skin incision about 0.5 cm was made, a 3.2 mm (130 mm length) diameter vertebral needle(guanlong, China) along the direction of 18G needle punctured. During operation, we used the preventive protection measures such as protective whole body aprons and lead collars, protective eye-glasses. In the process of operation, we used intermittent fluoroscopy. During active fluoroscopic monitoring, we didn’t need hold the puncturing needle, we could step away from the fluoroscope and stand behind the lead sheet. If the puncturing position and direction had deviation, after adjusted, we still stood behind the lead sheet.

Secondly, maximizing the distance from the x-ray tube will significantly reduce the exposure to operators. Whenever possible, the operator should step away from the patient during fluoroscopy. However, When the operator performs PVP with conventional manual cement delivery device, he has to stand beside the patient and can not be far away from the X ray tube because visualization of cement flow is crucial to optimize the results of this procedure. As stated previously, “every drop has to be monitored” [8,9]. In reality, most of the radiation dose received by the operators is consecutive to cement injection under continuous fluoroscopy. Nguyen-Kim Let al [10] reported an new injection device which is made up of a 30 cm long metallic tube in which the cement flows, propelled by a metallic mandrel. This mandrel is set with a handle which allows the user a firm grip to achieve high-pressure injection. Schis F et al. [11] used another new intraoperative injection system which acts like a gun to deliver the cement into the vertebral body through a cartridge connected to classical bone filler and separated from the gun itself by a distance of 1.2 m. the 1.2 m of extra distance from the fluoroscopic field acts to decrease the operators’ exposure. they demonstrated by comparasion with a classic injection group that the reduction is highly significant with a radiation dose reduction of greater than 80%. In our study we use another new cement delivery system, the handheld controller cable is 12 m, it can as long as possible therotically. during whole operation process, the radiation dose of group A was 0.10 μSv, group B was 12.09 μSv. a difference that was found to be statistically significant (P < 0.001) between group A and group B. the operator’s radiation dose was extremely significantly decreased in use of the new delivery system. In addition, we found that there is almost no learning curve required in order to master the new remote cement delivery system. The most important thing in use of it is below. When the bone cement reached the posterior wall or paravertebral venous plexus, we should immediately stop inject, at the same time, press the back button in order to release the injection pressure, so as to avoid the cement into the vertebral canal, intervertebral foramen and blood vessel.

In our study, to reduce the radiation exposure to patient and operators, we used the intermittent fluoroscopic monitoring, not only in needle-positioning, but also during cement injection. During cement injection, after injecting about 1 ml cement which can be seen from the display screen, because the volume of 3.2 *130 mm needle is about 1 ml, we took an image at every 0.2-0.4 ml through the forward key on the handheld controler. whenever cement was close to the posterior wall of the vertebral body, we took an image at only 0.1 ml. during the whole process, the radiation dose to the patient was 25.5 μSv, Radiation dose of patients is small. Of course, we can also use pulsed continuous fluoroscopy during cement injection, thus, we can “monitor every drop”. We will study the x-ray exposure in pulsed mode to patient and operator in the future.

## Conclusions

New cement delivery device plus meticulous preoperative preparation can significantly decrease radiation dose to operators.

## Competing interests

The authors declare that they have no competing interests.

## Authors’ contributions

GQZ analyzed the radiographic measurements, drafted the manuscript, and performed all surgery. YZG coordinated the research groups and conceived of the design of the study and participated in the study. SLC participated in the study. SD participated in the study. KG participated in the study. All authors read and approved the final manuscript.

## Pre-publication history

The pre-publication history for this paper can be accessed here:

http://www.biomedcentral.com/1471-2474/15/260/prepub

## Supplementary Material

Additional file 1 STROBE Statement. Click here for file
